# Long-term outcomes of older adults with acute COVID-19 following inpatient geriatric rehabilitation: a prospective cohort study from the Republic of Ireland

**DOI:** 10.1007/s11845-024-03723-4

**Published:** 2024-06-10

**Authors:** Aoife Mccarthy, Katie Robinson, Frances Dockery, Kara McLoughlin, Margaret O’Connor, Antonella Milos, Gillian Corey, Leonora Carey, Fiona Steed, Miriam Haaksma, Aoife Whiston, Audrey Tierney, Rose Galvin

**Affiliations:** 1 School of Allied Health, Faculty of Education and Health Sciences, Health Research Institute and Ageing Research Centre, University of Limerick, Limerick, Ireland; 2 Department of Geriatric and Stroke Medicine, and Integrated Care Team for Older People North Dublin, Beaumont Hospital, Dublin, Ireland; 3 Department of Ageing and Therapeutics, University Hospital Limerick, Limerick, Ireland; 4 School of Medicine, Faculty of Education and Health Sciences, University of Limerick, Limerick, Ireland; 5 UL Hospitals Group, University Hospital Limerick, Limerick, Ireland; 6 Department of Health, Baggot Street, Dublin, Ireland; 7 Coordinator EU-COGER Study, Department of Public Health and Primary Care, Leiden University Medical Center, Leiden, the Netherlands; 8 University Network for the Care Sector South-Holland, Leiden University Medical Center, Leiden, the Netherlands

**Keywords:** COVID-19, Inpatients, Patient reported outcome measures, Rehabilitation

## Abstract

**Background:**

There is a paucity of research reporting the long-term outcomes of older adults who have completed geriatric rehabilitation following COVID-19.

**Aim:**

The primary aim of this study is to describe the long-term functional outcomes of a cohort of older adults with acute COVID-19 who have completed inpatient geriatric rehabilitation.

**Methods:**

This is a subgroup analysis of Irish data from a pan-European prospective cohort study. Functional ability, patient reported symptoms, and quality of life were measured using the Barthel index, the COVID-19 Yorkshire Rehabilitation Screen, and the EQ-5D-5L, respectively.

**Results:**

Thirty patients enrolled in the study. The rate of mortality was 23.3% at 6 months after discharge from rehabilitation. Patients achieved a return to pre-admission functional ability but reported a significant increase in patient reported symptoms and their quality of life did not return to pre-admission levels when assessed at 6 months after discharge from rehabilitation.

**Conclusions:**

Multidisciplinary rehabilitation for older adults with acute COVID-19 infection can assist patients to return to their premorbid functional ability. On discharge from rehabilitation, ongoing follow-up of older adults is recommended to assist them to negotiate and manage ongoing symptomatology such as breathlessness or fatigue.

**Supplementary Information:**

The online version contains supplementary material available at 10.1007/s11845-024-03723-4.

## Introduction

COVID-19 is a contagious disease caused by the Sars-CoV-2 virus [1]. Acute illness can manifest with multisystem involvement, including the respiratory, cardiovascular, gastrointestinal, renal, neurological, ocular, cutaneous, musculoskeletal, haematological, and endocrine systems [2]. Disease presentation and severity has evolved since its emergence in 2019. Evidence suggests that the incidence of admission to intensive care and mortality was higher in wave one when compared to wave two in 2020 [3, 4]. In May, 2023, the World Health Organization (WHO) declared the end of the COVID-19 pandemic but described it as an established and ongoing health issue [5].

Older age [6–10] and the presence of frailty [11–13] are associated with increased risk of mortality and disease severity following COVID-19. Patients who are frail have a reduced physiological reserve associated with ageing which may impact their ability to respond to an acute multi-system disease [14]. This reduced physiological reserve is present in the endocrine, immune, cardiovascular, respiratory, and renal systems as well as the brain and skeletal muscles [14], many of which are also affected by acute viral COVID-19 [2]. Evidence suggests that older adults with acute COVID-19 infection progress to increased frailty states [15].

Geriatric rehabilitation is a multidimensional approach of diagnostic and therapeutic interventions, the purpose of which is to optimise functional capacity, promote activity, and preserve functional reserve and social participation in older people with disabling impairments [16]. Experts have highlighted the essential assessment and intervention domains when completing geriatric rehabilitation following COVID-19 [17] which includes completion of comprehensive geriatric assessment to address frailty, malnutrition, cognition, activities of daily living and participation, mood, pain and symptom management, retraining of mobility, strengthening exercises, psychological disturbances, and speech and swallow impairments and discharge planning to facilitate follow-up to the appropriate primary care or specialist outpatient care setting [17]. A systematic review by McCarthy and colleagues in 2023 [18] reported the outcomes of older adults who had completed multidisciplinary rehabilitation following acute COVID-19. Authors reported a significant improvement in functional ability following inpatient multidisciplinary rehabilitation (REM, SMD = 1.46, 95% CI 0.94 to 1.98); however, GRADE analysis revealed very low certainty of evidence across studies which limits the applicability of findings and patients were not followed up after discharge. Authors also highlighted that studies had not included all the recommended domains advocated by the European Geriatric Medicine Society [17] in the rehabilitation of older people with acute COVID-19 [18].

There has been considerable effort to describe long-term symptom presentation, the risk of multi-organ complications, and mortality and the predictive characteristics and associations for post-COVID-19 condition in older adults in the literature, particularly since 2022 [19–24]. Older adults are more likely to be symptomatic compared to their younger counterparts [20] and some of the most commonly reported long-term symptoms are fatigue, dyspnoea, cognitive difficulties, and pain [19–21, 24–26]. This evidence contributes to knowledge regarding disease presentation, prevalence, and evolution however does not evaluate the effectiveness of any potential interventions provided during the acute stage.

The primary aim of this study is to describe the long-term functional outcomes of a cohort of older adults with acute COVID-19 who have completed inpatient geriatric rehabilitation. A secondary aim is to describe changes in symptom presentation and quality of life (QoL) over a 6-month period following discharge from acute or sub-acute rehabilitation. This study is a subgroup analysis of Irish data gathered as part of a pan European project, the EU-COGER study. The EU-COGER study aimed to explore Activity of Daily Living outcomes, as well as other outcomes such as frailty and to describe the geriatric rehabilitation services provided to older adults post acute COVID-19 infection across Europe [27].

## Materials and methods

### Study design

This prospective cohort study is a subgroup analysis study of Irish data collected as part of a pan European project (EU-COGER), the protocol for which has been published elsewhere [27]. See Supplementary Material 1 for EU-COGER consortium list. The conduct and reporting of this study were in accordance with the STROBE checklist for cohort studies [28]. See Supplementary Material 2 for a copy of this checklist.

### Ethics approval

Ethical approval was obtained from the Health Service Executive Mid-Western Region Research Ethics Committee for University Hospital Limerick (UHL) on the 27th of April 2021 and amended approval was obtained on the 5th of August 2021 (025/2021) to include participation in the EU-COGER study. Ethical approval was obtained from Beaumont Hospital Ethics (Medical Research) committee on the 9th of April 2021 (21/31—COVID) and included approval to participate in the EU-COGER study also.

#### Participants

Recruitment to this study took place at two acute hospital sites in Ireland: Beaumont Hospital and University Hospital Limerick (UHL). Beaumont hospital (BH), located in Dublin city, provides emergency and acute care services to a largely urban population of approximately 290,000 people. UHL is an acute hospital in the Mid-West of Ireland serving an urban and rural population. It provides acute and emergency services to a region with a population of approximately 473,269 people.

Participants consecutively admitted to the acute hospital and who met the following inclusion criteria were recruited between April, 2021, and November, 2021:

- Recovering from acute COVID-19 infection requiring treatment in an acute hospital, confirmed with polymerase chain reaction for viral RNA, serology for antibodies against SARS-CoV-2, or an approved alternative test.
- Accepted for geriatric rehabilitation following acute COVID-19 infection in the hospital setting.

Due to the study design, study sample size calculation was inappropriate and the decision to recruit over the course of 6 months was in keeping with the recruitment period for the EU-COGER study. Patients from UHL were recruited by a clinical research nurse (GC) and patients from BH were recruited by a geriatrician (FD) and an occupational therapist (KMcL).

Patients were excluded if they had a severe cognitive impairment that would impact their decision-making ability to participate in a study and/or those who did not provide written informed consent. The presence of a severe cognitive impairment was determined by a comprehensive chart review by the clinical research nurse and deemed positive if the patient met any one of the following criteria:

- Montreal cognitive assessment score of ≤ 10.
- Documented diagnosis of middle- to late-stage dementia.

The interventions provided were determined by each patients’ unique health and rehabilitation needs which included, but was not limited to Occupational Therapy, Physiotherapy, Clinical Nutrition and Dietetics, and Specialist Nursing.

### Procedures and outcome measures

A comprehensive list of study variables and time points can be found in the EU-COGER study protocol [27]. Outcomes reported for the purpose of this secondary analysis are as follows: demographics and baseline clinical characteristics including age, sex, pre-morbid residence, date of admission, medical history and co-morbidities, date of admission, date of discharge, acute hospital length of stay, number of days engaged in rehabilitation, discharge destination, and COVID-19 status including testing method.

Study timepoints for clinical outcomes were as follows:

- T0: pre-morbid (retrospective evaluation, where applicable)
- T1: on admission to rehabilitation
- T2: on discharge from rehabilitation
- T3: 6 weeks after discharge from rehabilitation (obtained via telephone interview)
- T4: 6 months after discharge from rehabilitation (obtained via telephone interview)

For this study, pre-morbid ratings refer to performance/presentation before acute viral illness with COVID-19. All UHL data were gathered by the study’s first author (AMcC) and a clinical research nurse (GC). All BH data were gathered by the EU-COGER national coordinator for Ireland who was also a geriatrician (FD) and an occupational therapist (KM).

Functional ability was measured through use of the Barthel index [29] given its ability to measure change in function in the older adult population [30]. The Barthel is a 10-item ordinal instrument reporting patient’s performance of activities of daily living, including bathing, toileting, and dressing. The Barthel was administered at all timepoints (T0–T4).

Patient reported symptoms were measured through use of the self-report COVID-19 Yorkshire Rehabilitation Screen (C-19 YRS). The original version of the screen was used [31]. The C-19 YRS is a patient-reported outcome measure and was developed by healthcare professionals in the UK’s National Health Service who were practicing in the rehabilitation of patients with COVID-19. The scale asks patients to rate their perceived symptom severity or functional difficulty “now” and “pre-COVID” in the domains of breathlessness, mobility, fatigue, personal care, usual activities, pain/discomfort, anxiety, depression, and global perceived health status. New onset symptoms pertaining to laryngeal complications, voice, swallowing, nutrition, continence, cognition, and communication are also recorded. The perceptions of family and care givers regarding symptomatology and presentation are also sought. Ordinal items of the C-19 YRS are rated on a Likert scale of 0–10. Zero indicates no difficulty/absence of symptoms and 10 indicates severe difficulty/severe symptoms, except for global perceived health where higher ratings indicate better health and lower ratings indicate poorer health. Categorical items are rated with a yes/no response. No overall score can be generated in this earliest version of the screen. The C-19 YRS was administered at T2 and T4. Five participants had the assessment of the C-19 YRS at T3 and T4.

QoL was measured through use of the EQ-5D-5L [32]. The tool comprises of five domains: mobility, self-care, usual activities, pain/discomfort, and anxiety/depression. The respondent rates each domain on five levels: no problems, slight problems, moderate problems, severe problems, and extreme problems. The tool also requires respondents to rate their overall health on a visual analogue scale. The tool generates a profile score based on the respondents rating of the five domains. From the profile score, an index score can be generated based on country-specific tariffs. The derived index scores range from −0.59 to 1. The maximum index score of 1 indicates full health and lower scores indicate poorer health. The EQ-5D-5L was administered at all timepoints (T0–T4).

### Statistical analysis

Descriptive statistics were used to profile the baseline characteristics of the cohort. Categorical measures, for example gender, were analysed in terms of frequencies and percentages; continuous measures, for example Barthel index, were analysed in terms of means and standard deviations.

#### Function and quality of Life

Separate repeated measures ANOVAs were conducted to explore differences in Barthel index scores and EQ-5D-5L index scores across the following time points: T0, T1, and T4. Data from these three timepoints were used given the focus of our research question on long-term outcomes. Where necessary, violations of sphericity resulted in use of a greenhouse Geisser correction. All analyses were conducted using JASP v.0.16.2. Post-hoc analysis was also carried out, if analysis of variance demonstrated a significant result, to determine the significance of paired outcomes between timepoints. Data were graphically represented using raincloud plots that combine a cloud of data points with a box plot and a one-sided violin plot.

#### Patient-reported symptoms

Paired samples *t*-test or their non-parametric equivalent—Wilcoxon matched pairs signed rank tests—was conducted to examine C19-YRS ratings “now” and “pre-COVID” on the following domains: breathlessness at rest, dressing, and stairs, problems with walking, personal care, and usual activities, fatigue, pain, anxiety, depression, and overall health.

## Results

### Demographic, process, and clinical characteristics

Thirty patients enrolled in the study. Fifteen patients were female (50%). The mean age of the sample was 74.4 years (SD 11.8 years). All older adults had co-morbidities prior to acute COVID-19 viral illness, and all resided in their own home. Nine older adults (30%) required an admission to ICU during their acute hospital stay with a median length of ICU stay of 25.5 days (IQR = 31.75). The median length of stay in rehabilitation was 30.5 days (IQR = 30.5).

On discharge from rehabilitation, 90% of patients were discharged to their own home (*n* = 27). At 6 weeks from discharge, the incidence of mortality was 3.3% (*n* = 1) and at 6 months this had increased to 23% (*n* = 7). At 6 months, a further three participants were also lost to follow-up or declined to take part any further in data collection but consented for data gathered to date to be used. Data analyses for long-term outcomes were completed for 20 participants. See Table 1 for summary of patient characteristics.

**Table 1:** Patient characteristics (*N* = 30)

**Characteristics**	**Value**
Long term outcome analysis, *n* (%)	20 (66.7)
Gender, female, *n* (%)	15 (50)
Age, mean (SD)	74.4 (11.8)
Patients with co-morbidities, *n* (%)	30 (100)
ICU admission, *n* (%)	9 (30)
ICU LOS, days, median (IQR)	25.5 (31.8)
Rehabilitation LOS, days, median (IQR)	30.5 (30.5)
Discharged home from rehabilitation, n (%)	27 (90)
6 week mortality, *n* (%)	1 (3.3)
6 month mortality, *n* (%)	7 (23.3)

*ICU* intensive care unit, *LOS* length of stay

### Functional ability

**Fig. 1:**
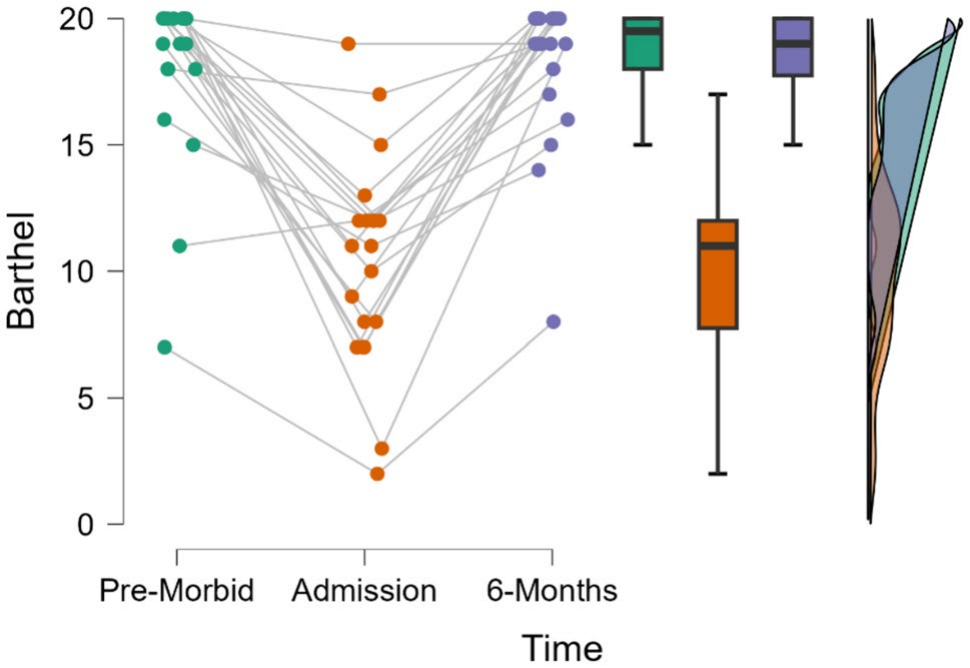
Raincloud plot for Barthel scores

A significant difference was observed in Barthel index scores across timepoints—T0, T1, and T4, *F*(1.24, 24.47) = 55.22, *p* < 0.001, *n*^2^ = 0.744. Post-hoc tests showed a significant decrease in Barthel scores when comparing T0 (*M* = 18.00, *SD* = 3.45) and T1 measures (*M* = 10.35, *SD* = 4.18), with a mean difference (MD) of 7.650 (*p* < 0.001). A significant increase was observed when comparing T1 and T4 measures (T4 *M* = 18.10, *SD* = 2.97) with MD of 7.750. No significant difference was observed when comparing T0 and T4 measures with a MD of 0.100 (*p* = 0.907). This suggests a return to pre-morbid functioning at 6 months following engagement in geriatric rehabilitation. See Fig. 1 for raincloud plot demonstrating this change over time. See Table 2 for post-hoc data for functional ability.

**Table 2:** Functional ability (Barthel Index) across timepoints

	Pre-morbid	Admission	6 months	
Barthel Index score Mean (SD)	18 (3.45)	10.35 (4.18)	18.10 (2.97)	

		Mean difference (MD)	SE	*p*-value
Pre-morbid	Admission	7.650	0.846	< 0.001
	6 months	0.100	0.846	0.907
Admission	6 months	7.750	0.846	< 0.001

### Patient reported symptoms

There was a significant increase in nine of the C-19 YRS domains, indicating a worsening of symptoms/presentation from preadmission to 6 months after discharge from rehabilitation. No significant difference was observed for the domain “breathlessness at rest” and “pain”. See Table 3 for summary of C-19 YRS data analysis.

**Table 3:** C-19 YRS outcomes

**C19-YRS domain**		**M**	**SD**	**Difference**	**W**	***p***
**Breathlessness at rest**	Pre-COVID	0.10	0.56	0.69	3.000	0.279
	Now	0.79	1.78			
**Breathlessness dressing**	Pre-COVID	0.38	1.40	1.99	3.500	0.016
	Now	2.37	2.95			
**Breathlessness stairs**	Pre-COVID	0.85	2.41	3.28	0.000	0.004
	Now	4.13	3.38			
**Walking problems**	Pre-COVID	1.03	2.35	2.08	0.000	0.001
	Now	3.11	2.54			
**Fatigue**	Pre-COVID	0.76	1.62	3.4	0.000	0.001
	Now	4.16	3.25			
**Personal care problems**	Pre-COVID	1.45	2.91	1	1.000	0.008
	Now	2.45	2.87			
**Usual activity problem**	Pre-COVID	1.69	3.40	2.66	0.000	0.004
	Now	4.35	2.08			
**Pain**	Pre-COVID	1.61	2.94	1.69	0.000	0.059
	Now	3.30	3.85			
**Anxiety**	Pre-COVID	0.69	1.49	2.61	0.000	0.020
	Now	3.30	2.21			
**Depression**	Pre-COVID	0.39	0.83	1.91	0.000	0.009
	Now	2.30	2.87			
Overall health*	Pre-COVID	8.72	1.56	1.47	120.000	< 0.001
	Now	7.25	2.05			

*Pre-COVID* patient’s retrospective rating of symptoms/function prior to contracting COVID-19, *Now* patient’s rating of current symptoms/function

*For overall health, higher ratings indicate better health, and lower ratings indicate poorer health

### QoL

Repeated measures ANOVA showed a significant difference in EQ5D5L index scores across timepoints T0, T1, and T4, *F*(2, 34) = 19.22, *p* < 0.001, *n*^2^ = 0.531. Post-hoc tests showed a significant decrease in QoL when comparing T0 (*M* = 0.878, *SD* = 0.137) and T1 measures (*M* = 0.380, *SD* = 0.350) with a MD of 0.498 (*p* < 0.05). A significant decrease was observed when comparing T0 and T4 (T4: *M* = 0.653, *SD* = 0.300) measures with a MD of 0.225. A significant increase was also observed when comparing T1 and T4 measures with a MD of 0.273.

**Fig. 2:**
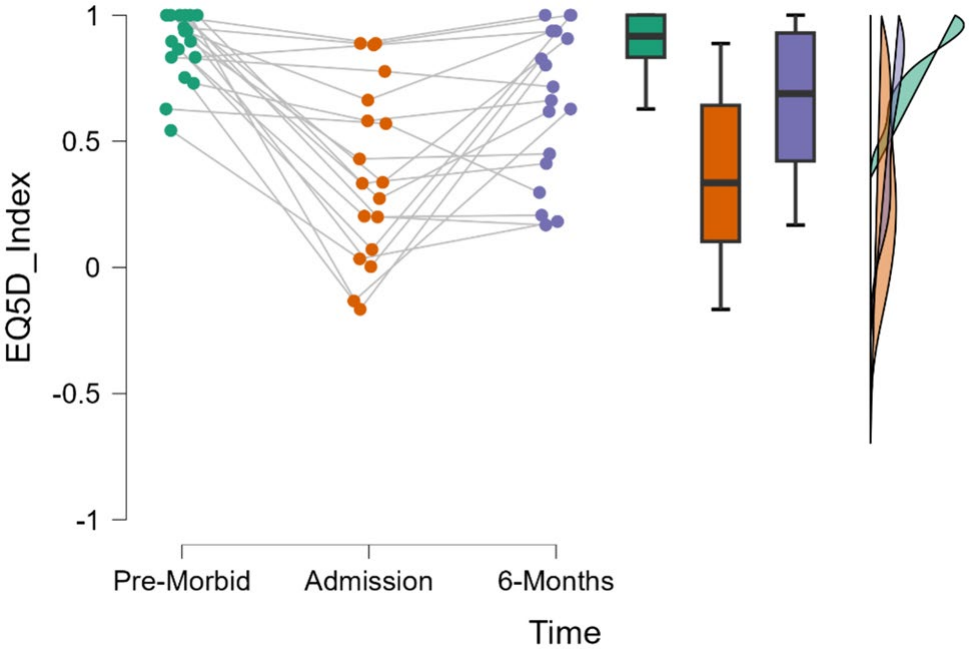
Raincloud plot for EQ-5D-5L

This suggests that although QoL at 6 months does not return to pre-morbid levels, it has significantly improved when compared to admission to rehabilitation. See Fig. 2 for raincloud plot demonstrating this change over time. See Table 4 for post-hoc data for EQ-5D-5L.

**Table 4:** Quality of life (EQ-5D-5L) across timepoints

	Pre-morbid	Admission	6-months	
EQ-5D-5L scores Mean (SD)	EQ-5D-5L scores	0.38 (0.35)	0.653 (0.3)	

		Mean difference (MD)	SE	*P* value
Pre-morbid	Admission	0.498	0.080	< 0.001
	6 months	0.225	0.080	0.008
Admission	6 months	0.273	0.080	0.004

## Discussion

This is the first Irish cohort study to prospectively follow older adults with COVID-19 who have undergone a period of inpatient multidisciplinary rehabilitation. Thirty patients enrolled in the study across two sites. There was a relatively high mortality rate of 23.3% at 6 months after discharge from rehabilitation. Results suggest that patients achieved a return to pre-admission functional ability when assessed at 6 months after discharge from rehabilitation. However, patients reported worsening of symptoms from pre-admission to 6 months after discharge from rehabilitation. Patient-reported QoL did not return to pre-admission level however significantly improved between admission to rehabilitation and 6 months post-discharge.

A key finding of this cohort study is that there was no significant difference between premorbid functional ability and functional ability 6 months post-discharge, indicated by post-hoc analysis. This finding suggests patients achieved their pre-admission ability to complete and engage in activities of daily living. Existing evidence from Sathyamurthy and colleagues (2021) supports this finding. They reported no significant difference between mean activity of daily living and instrumental activities of daily living at 90 days post-discharge from acute hospital admission for older adults with mild to severe COVID-19. The presence, nature, and extent of rehabilitative interventions, if any, were not described in this study [33]. There is also contradictory evidence which reports the prevalence of functional impairments in older adults and explores associations between COVID-19 and decreased independent functioning [34]. Bae and colleagues reported a significant association between COVID-19 and decreased independence in activities of daily living (RR: 1.47, 95%CI 1.1–1.96, *p* = 0.0079); however, it was unclear at what time outcomes were gathered and data was gathered from a population study with unknown treatment and/or therapy interventions provided to patients.

The majority of C-19 YRS domains indicated a worsening of symptoms from preadmission to 6 months after discharge from rehabilitation. There is a large body of evidence describing patient-reported symptoms in non-interventional studies. These studies report prevalence of at least one ongoing patient-reported symptom of 60 to 83% [19, 20, 24–26]. In this study, the rate was 95%. The study timepoints varied from 30 days post-acute infection to up to 6 months post-infection [19, 20, 24]. In some cases, the exact follow-up time was not clear [25, 26]. Our finding supports the existing body of evidence that older people following acute COVID-19 infection present with ongoing and long-term persistent symptomatology.

A key finding of this cohort study is that patients did not return to their premorbid QoL; however, it was significantly improved at 6 months following discharge from rehabilitation when compared to admission to rehabilitation. Similar to our findings, a systematic review found that irrespective of time lapsed since discharge from hospital or recovery, COVID-19 patients' QoL was significantly affected [35]. Similarly, a study with Iranian older adults published after this systematic review found lower self-rated QoL (80.15 ± 14.85 on World Health Organization Quality of Life Questionnaire) among older adults with a history of COVID-19 infection when compared to those without a history of COVID-19 (85.25 ± 14.09) however time since infection was not reported [36]. Contradictory findings have also been reported. Salci and colleagues [21] reported that overall average QoL was high among a sample of older adult Brazilians with long COVID syndrome (n=403 and 79.6% of older adults rated their QoL as high). Importantly, older age is associated with poorer QoL outcomes post COVID-19 infection [35] , alongside other factors such as female sex, the presence of comorbidities, intensive care unit (ICU) admission, prolonged ICU stay, and the need for mechanical ventilation. Potential targets for intervention to improve QOL among older adults after COVID-19 infection include addressing factors shown to negatively impact QOL in this group such as fatigue, pain, low physical activity and cognitive-communication problems [19].

This study presents promising outcomes to support multidisciplinary rehabilitation for older adults with COVID-19 in terms of patients’ return to premorbid functioning. It does however also highlight patients need for ongoing support following discharge from acute hospitals and rehabilitation units to assist them to negotiate and manage some long-term symptoms that have the potential to impact function and quality of life. International guidance supports the ongoing monitoring of patients at 6 weeks and 6 months [17] after acute COVID-19 infection, and our results reiterate the need for such services. This can be provided at an outpatient, community, or integrated service. This research also highlights the importance of developing clinical care pathways and models of care for older adults with post-COVID-19. Existing guidance is limited to the domains for assessment during follow-up [17]. It lacks specific recommendations in relation to the appropriate setting, frequency of intervention, team composition, and model of care. Research regarding models of care for post-COVID-19 condition exists but is not specific to the unique needs of an older adult [37].

We know that older adults are at risk of progressing to worsened frailty states following acute COVID-19 infection [15] and that COVID-19 can exacerbate existing chronic health conditions, for example, diabetes [38]. These assertions and the findings of this cohort study support the need for future research to describe and evaluate community, and/or outpatient-based services for older adults to address the long-term complications of acute COVID-19 infection and to evaluate their effectiveness. Identifying post-COVID-19 deficits in older adults and distinguishing them from frailty syndromes and the presence of multimorbidity is challenging.

The results of this study are generalisable to our cohort of interest, older adults with COVID-19. The patients recruited were of representative age and patients were recruited from hospitals with a mix of urban and rural populations.

### Limitations of the study

A limitation of this study is the small sample size. Patients with severe cognitive impairment were also excluded which limits the applicability of results to this subgroup of the population. An important point to consider, when interpreting the results presented by our study, is the fact that outcome measures used, although valid and reliable, are patient reported in nature. Patient-reported measures have limitations in terms of content and face validity, impact of bias, and they are vulnerable to the influence of factors such as pain, psychological distress, and social factors [39].

The Barthel index, although appropriate for use with the older adult population, is designed to reflect performance in personal and basic activities of daily living. Instruments designed to include higher-level activities of daily living such as the Functional Independence Measure/Functional Assessment Measure [40] may provide a more robust evaluation of overall function. Logistical and resource limitations influenced the researcher’s capacity to complete such tools as they cannot be completed over the phone and require prolonged evaluation. This highlights the need for future research to include objective and face-to-face evaluation of function which includes more detailed measures for functional ability.

## Conclusion

Multidisciplinary rehabilitation for older adults with acute COVID-19 infection can assist patients to return to baseline functional ability; however, ongoing follow-up and monitoring is required to assist patients to negotiate and manage ongoing symptomatology.

## Supplementary Information

Below is the link to the electronic supplementary material.

Supplementary file1 (DOCX 18 KB)

Supplementary file2 (DOCX 32 KB)

## Data Availability

The data associated with this paper are not publicly available but are available from the corresponding author on reasonable request.
