# Supplementary material for: Long-term outcomes of older adults with acute COVID-19 following inpatient geriatric rehabilitation: a prospective cohort study from the Republic of Ireland
Source: Ir J Med Sci. 2024 Jun 10;193(5):2567–75. doi: 10.1007/s11845-024-03723-4 (PMC11450069; doi:10.1007/s11845-024-03723-4)
Supplement: Supplementary file 1 — Supplementary file1 (DOCX 18 KB) [file 11845_2024_3723_MOESM1_ESM.docx]

Supplementary material 1: EUCOGER consortium list

| **Name of health centre** | **Country** | **Study coordinator #1** | **Study coordinator #2** | **Study coordinator #3** |
| --- | --- | --- | --- | --- |
| Vseobecna fakultní nemocnice | Czech Republic | Eva Topinková | Lucie Bautzká | Helena Michaálková |
| Agaplesion Bethanien Hospital | Germany | Stefan Grund | Thomas Mross | Lotte Feesche |
| Robert-Bosch-Krankenhaus | Germany | Rebekka Leonhardt | Clemens Becker |  |
| Geriatrisches Zentrum Karlsruhe | Germany | Jan Gerhardus | Brigitte R. Metz |  |
| Geriatrische Rehabilitationsklinik Diakonissenkrankenhaus Mannheim | Germany | Diana Franke-Chowdhury |  |  |
| University of Limerick Hospital Group (ULHG) | Ireland | Rose Galvin | Aoife McCarthy |  |
| Beaumont Hospital | Ireland | Frances Dockery | Kara McLoughlin |  |
| Fliman geriatric rehabilitation center | Israel | Bahaa Francis |  |  |
| IRCCS Istituti Clinici Maugeri | Italy | Matteo Cesari | Annalisa Valentini |  |
| Karin Grech Hospital | Malta | Mark Vassallo | Maria Bonnici |  |
| Russian Clinical and Research Center of Gerontology | Russia | Olga Nikolaevna Tkacheva | Ksenia Eruslanova |  |
| Moscow Rehabilitation center | Russia | Luba Matchekhina |  |  |
| Parc Sanitari Pere Virgili | Spain | Laura Monica Perez Bazan |  |  |
| Hospital Universitari Sant Joan de Reus | Spain | Esther Roquer Fanlo |  |  |
| Hospital Universitari Parc de Salut Mar | Spain | Anna Renom Guiteras | Lizzeth Angela Canchucaja |  |
| Hospital Central de la Cruz Roja San José y Santa Adela | Spain | Beatriz Pallardo | Sergio Martínez Zujeros |  |
| Hospital San Joan de Deu Mallorca | Spain | Margarita Viñuela | Oriol Miralles Resina |  |
| Hospital Guadarrama | Spain | Gema Isabel Dominguez | Sarah Caro Bragado |  |
| Hospital de Barcelona | Spain | Nadia Stasi | Jennifer Garrillo Cepeda |  |
| Consorci Sanitari Alt'Pènedes i Garraf | Spain | Marta Arroyo-Huidobro | Ana Gonzalez |  |
| Leiden University Medical Center | the Netherlands | Wilco Achterberg | Monique Caljouw | Miriam Haaksma |
| Omring | the Netherlands | Saskia Drijver |  |  |
| Zorgcirkel | the Netherlands | Paula Vonk |  |  |
| BrabantZorg | the Netherlands | Liesbeth Sikken | Irma Baars |  |
| Ijsselheem | the Netherlands | Nathalie Deden |  |  |
| Topaz Revitel | the Netherlands | Gerda Nijgh | Sylvia van der Drift |  |
| Tante Louise | the Netherlands | Heike de Wever | Els Calle |  |
| MUMC+\|Herstelzorg - Vitala+ | the Netherlands | Kaoutar Karramass | Josette Hendriks |  |
| Axion continu | the Netherlands | Lauren Ebbes |  |  |
| TriviumMeulenbeltZorg Almelo | the Netherlands | Anne Hartman | Hatice Koc |  |
| TriviumMeulenbeltZorg Hengelo | the Netherlands | Laura de Vries |  |  |
| Patyna | the Netherlands | Hylco Bouwstra |  |  |
| Careyn | the Netherlands | Laura Langendoen-Wigman |  |  |
| Sensire | the Netherlands | Berber Oldenbeuving | Sabine Noordam-Hemeltjen |  |
| Azora | the Netherlands | Liesbeth Lanting | Lulu Andela |  |
| Argos Zorggroep | the Netherlands | Mathilde Meerkerk |  |  |
| Meriant (Alliade) | the Netherlands | Lianne Willemstein | Krisztina Krasznai |  |
| Liemerije | the Netherlands | Janneke Wolting |  |  |
| Laurens Intermezzo Zuid | the Netherlands | Janette Tazmi |  |  |
| de Wever | the Netherlands | Eveline Keustermans |  |  |
| Icare - De Boshof | the Netherlands | Janetta de Vries | Sanne van Weers |  |
| SVRZ 't Gasthuis | the Netherlands | Lenni Boogaard |  |  |
| De Betuwe, Zorgcentrum Beatrix | the Netherlands | Simone Been |  |  |
| Archipel Zorggroep | the Netherlands | Danielle Termeer |  |  |
| Florence | the Netherlands | Patricia te Pas | Eva Lodewijks |  |
| Pieter van Foreest, locatie Bieslandhof | the Netherlands | Jeroen van den Berg |  |  |
| Reactiveringscentrum Klimop | the Netherlands | Sandra Prent | Marloes Boontje |  |
| Zorgspectrum Nieuwegein | the Netherlands | Joël Harms | Jeffrey Bakker |  |
| Zorggroep Maas en Waal | the Netherlands | Carolien de Croon |  |  |
| Attent | the Netherlands | Christa van Schieveen |  |  |
| Vivium Flevoburen (Zorggroep Almere) | the Netherlands | Ewout Smit |  |  |
| Kennemerhart Schoterhof | the Netherlands | Patricia van Berlo |  |  |
| Van Neynsel | the Netherlands | Dionne Ruchtie |  |  |
| Sheffield teaching Hospitals | UK | Jane Manson |  |  |
| Frimley Health NHS Foundation Trust | UK | Maria Espasandin | Lucy Abbott |  |
| Harrogate District Hospital | UK | Sarah Chadwick | Rebecca Watts |  |
| Imperial College Healthcare NHS Trust | UK | Melani Dani | Jackie McNicholas |  |
| University Hospitals of Derby and Burton | UK | Adam Gordon |  |  |
| Calderdale & Huddersfield | UK | Vincent Chau |  |  |
| Derbyshire Community Health Services | UK | Andy Cole |  |  |
